# Systematic review and meta-analysis of the current literature on tocilizumab in patients with refractory Takayasu arteritis

**DOI:** 10.3389/fimmu.2023.1084558

**Published:** 2023-02-08

**Authors:** Limei Kang, Yang Liu, Zhongling Luo, Yueyuan Zhou, Bo Chen, Geng Yin, Qibing Xie

**Affiliations:** ^1^ Department of Rheumatology and Immunology, West China Hospital, Sichuan University, Chengdu, China; ^2^ Department of Vascular Surgery, West China Hospital, Sichuan University, Chengdu, China; ^3^ Department of General Practice, General Practice Medical Center, West China Hospital, Sichuan University, Chengdu, China

**Keywords:** tocilizumab, Takayasu arteritis, refractory, baseline characteristic, clinical outcome

## Abstract

**Objective:**

To present the pooled quantitative evidence of baseline characteristics and clinical outcomes of tocilizumab (TCZ) in patients with refractory Takayasu arteritis (TAK).

**Methods:**

A comprehensive systematic review and meta-analysis was performed on all available studies retrieved from the MEDLINE, Embase, and Cochrane databases, using TCZ in patients with refractory TAK. We applied the commands *metan* and *metaprop_one* in Stata Software to pool overall estimates of continuous data and binomial data, respectively. A random-effects model was recruited for analysis.

**Results:**

Nineteen studies with 466 patients were included in this meta-analysis. The mean age at implementation of TCZ was 34.32 years. Female sex and Numano Type V were the most prominent baseline characteristics. During the 12-month follow-up when receiving TCZ treatment, pooled CRP was 1.17 mg/L (95% confidence interval [CI] -0.18-2.52), pooled ESR was 3.54 mm/h (95% CI 0.51-6.58), and pooled glucocorticoid dose was 6.26 mg/d (95% CI 4.24-8.27). Approximately 76% (95% CI 58-87%) of patients achieved a decrease in glucocorticoid dosage. Meanwhile, patients with TAK had a remission rate of 79% (95% CI 69-86%), a relapse rate of 17% (95% CI 5-45%), an imaging progress rate of 16% (95% CI 9-27%), and a retention rate of 68% (95% CI 50-82%). Adverse events occurred in 16% (95% CI 5-39%) of patients, and infection was the most common adverse event, with a rate of 12% (95% CI 5-28%).

**Conclusion:**

TCZ treatment can provide favorable outcomes in terms of inflammatory markers, steroid-sparing effects, clinical response, drug retention and minimizing adverse effects for patients with refractory TAK.

## Introduction

Takayasu arteritis (TAK), a type of large-vessel vasculitis, is defined as an immune arteritis involving granulomatous inflammation of the aorta and its major branches (1). It is predominantly a disease of young Asian women, with variable clinical manifestations ranging from asymptomatic incidental discovery to fever, myalgias, hypertension, limb claudication and absent pulses due to arterial stenosis and/or aneurysms (1). The standard first-line treatment option for active TAK patients is high-dose glucocorticoids (GCs) plus adjunctive conventional synthetic disease-modifying antirheumatic drugs (csDMARDs), as recommended by both EULAR and ACR guidelines (2, 3). However, approximately 40% of TAK patients can’t control the disease despite high-dose GC therapy plus csDMARDs (4, 5). Meanwhile, patients frequently relapse during GC tapering, with reported rates ranging from 50–80% in the literature (6, 7). How to treat these refractory TAK patients remains an unresolved issue.

Inflammation plays a crucial role in the pathophysiology of TAK (1). Inflammatory cells, including T-helper 1 and T-helper 17 cells, and proinflammatory cytokines, including interferon-γ, tumor necrosis factor-α, and interleukin-6 (IL-6), are involved in the initiation and propagation of inflammation in TAK (8–10). Hence, biologic targeted therapies should be considered as an alternate choice. To date, tumor necrosis factor-α inhibitors (TNFIs), such as infliximab and its biosimilar, have been shown to improve remission and relapse in several observational studies (11–15), and are recommended to refractory TAK patients by ACR guidelines (3). However, tocilizumab (TCZ), an anti-IL-6 receptor that is efficacious for active giant cell arteritis (GCA) (16, 17), is not recommended for refractory TAK patients due to a lack of forceful clinical evidence (2, 3). In the only randomized controlled trial (RCT) of TCZ in refractory TAK patients, the results from both intent-to-treat and per-protocol set populations showed no statistically significant difference in terms of relapse-free survival between TCZ and placebo (18). Data from other case series or retrospective cohorts presented some conflicting results, with a wide range of relapse rates from 0% to 66.7% during 12-month follow-up (18–21). Most studies had a small patient sample size, limiting the application of findings to the actual population and clinical practice.

Recently, a series of studies concerning efficacy and safety of TCZ in refractory TAK patients have been published (19–29). This systematic review and meta-analysis presents pooled quantitative evidence of baseline characteristics and clinical outcomes of TCZ in refractory TAK patients.

## Materials and methods

This systematic review and meta-analysis was performed according to the Cochrane Handbook for Systematic Reviews of Interventions (version 6.3) (30) and the Preferred Reporting Items for Systematic Reviews and Meta-Analyses (PRISMA) statement (31).

### Search strategy

The MEDLINE, Embase, and Cochrane databases were searched through Ovid access (https://ovidsp.ovid.com). The following keywords or Medical Subject Headings terms were used: “tocilizumab [Mesh]” or “IL-6 [Mesh]” or “tocilizumab [All Fields]” or “IL-6 [All Fields]” or “inerleukin-6 [All Fields]”; “arteritis [Mesh]” or “vasculitis [Mesh]” or “arteritis [All Fields]” or “vasculitis [All Fields]”; and “takayasu [All Fields]”. There was no restriction on publication dates, but publications were limited to the English language. Potential related studies in the reference lists of included studies were manually searched one at a time. The latest search was updated on July 18, 2022. The detailed search strategies are listed in **Supplementary Table 1**.

### Selection criteria

A preestablished selection criteria was used to select candidate studies by two independent authors (LK and YL), and any discrepancies were discussed and solved by a third author (ZL). Inclusion criteria were as follows: clinical studies that used TCZ in patients with refractory TAK; reported baseline characteristics or clinical outcomes; and used a sample size of ≥ 5 patients. Exclusion criteria were as follows: reviews, letters, or conference abstracts; efficacy and safety studies of TCZ in newly diagnosed or treatment-naive TAK; studies that reported other biological DMARDs, such as infliximab, etanercept, adalimumab, etc.

### Data extraction

Two independent authors (LK and YL) extracted available data from all included studies. A third author (ZL) would intervene if there were any disagreements. Extracted data were listed as follows when available: author, year, country, study design, patient source, trial period, sample size, gender, age, diagnosis criteria, Numano classification, onset age, disease duration, previous DMARDs, TCZ usage, TCZ duration, follow-up, and clinical outcomes (including serological response, clinical response, imaging response, drug retention, and adverse events). In addition, some necessary data were retrieved by emailing the authors if data were inadequate to pool effect estimates.

### Definition

Definitions of the following terms were based on the recommendations of EULAR and ACR guidelines (2, 3). Specifically, refractory TAK was defined as a persistent active disease associated with an inability to induce remission despite an appropriate course of standard care therapy. Remission was defined as absence of all clinical signs and symptoms attributed to active TAK and normalization of the C-reactive protein (CRP) and erythrocyte sedimentation rate (ESR), with or without immunosuppressive therapy. Relapse was defined as the recurrence of active disease following a period of remission. Imaging progress was defined as the progress of vessel wall thickness, stenosis, occlusions, aneurysms, or new vascular lesions at the follow-up imaging (28). Drug retention was defined as the duration from the time of administration to the time of definitive TCZ interruption (22).

### Quality assessment

Quality assessments of included studies were performed using the Newcastle‒Ottawa Scale (www.ohri.ca/programs/clinical_epidemiology/oxford.asp) for cohort studies, and the 20-criterion quality appraisal checklist with the modified Delphi technique (32) for case reports and case series by two independent authors (LK and YL). Details of the quality assessment are presented in **Supplementary Tables 2–4**.

### Statistical analysis

All statistical analyses were performed according to the Cochrane Handbook for Systematic Reviews of Interventions (version 6.3) (30).

For continuous variables, reported means and standard deviations were directly extracted from individual studies; if they were unavailable, means and standard deviations were obtained using the methods introduced by Wan and Luo et al. (33, 34) Overall estimates were pooled in Stata Software (version 15.1, Stata Corporation, College Station, TX, USA) by applying the command *metan*. For binomial data, numbers of events and totals for all studies were extracted, and study-specific proportions with 95% confidence intervals (CIs) were computed using the exact method (35). Considering the significant between-study heterogeneity and specific estimates with cure rates at or close to 0% or 100% in some studies, the logistic-normal random-effect model was employed to calculate the pooled estimates with the updated command *metaprop_one* in Stata Software (35). With this model, overall pooled proportions with 95% CIs were obtained by logit transformation and back transformation. The *Chi* ^2^ statistic of the likelihood ratio (LR) test that compares the random-effect and fixed-effect models was presented to explore potential heterogeneity across studies, which was analogous to the Q-statistic. In this review, *p* < 0.05 was considered significant.

## Results

### Included studies

**Figure 1:**
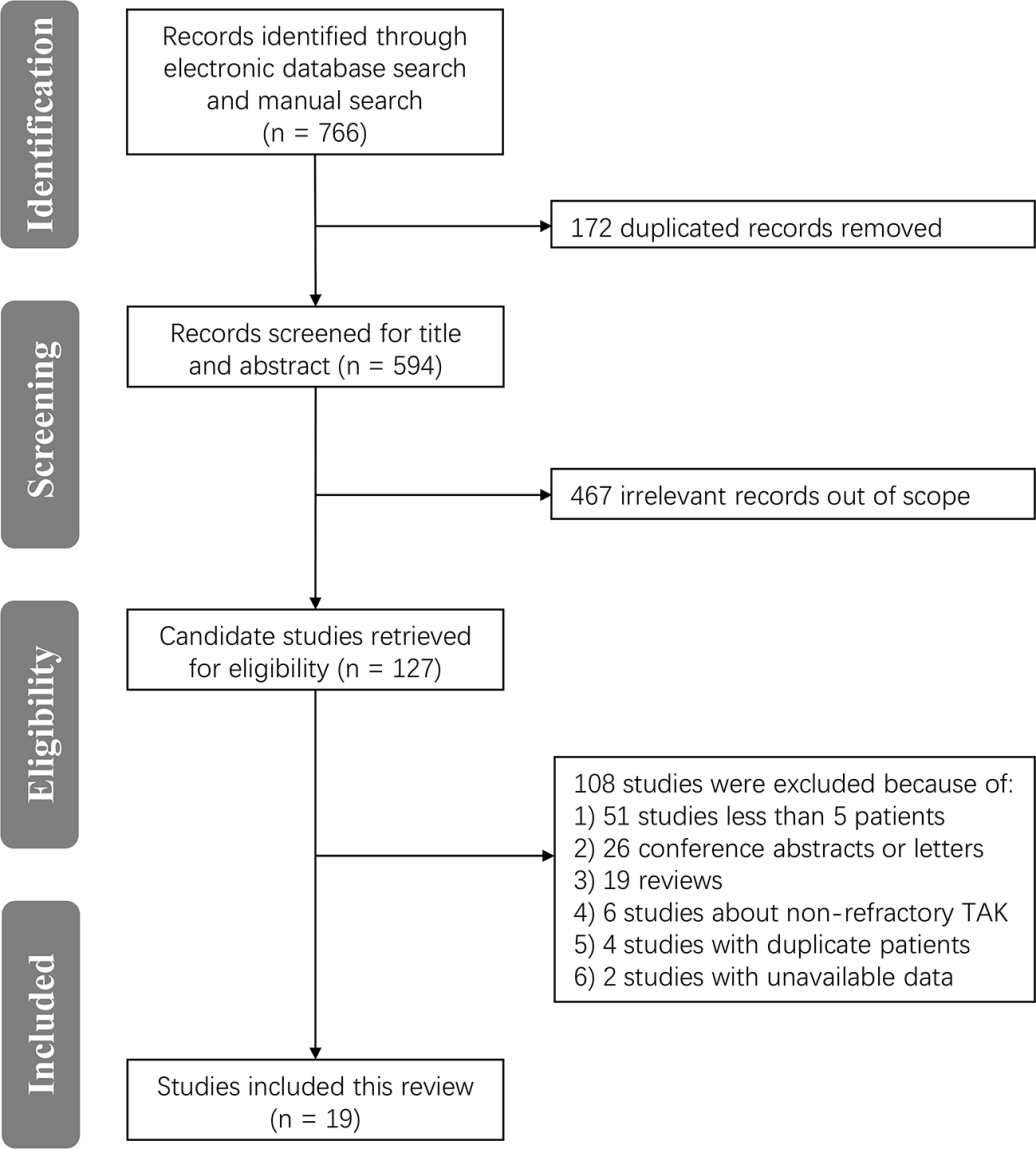
PRISMA flow chart of the literature search and selection of included studies.

In the latest update, a total of 766 potentially relevant records were identified in a MEDLINE, Embase, and Cochrane database search. After removing duplicates and screening titles and abstracts, 127 candidate publications were retrieved for further eligibility assessment and were read in full. Of these, 108 studies were excluded due to a sample size of less than five patients, conference abstracts, letters, non-refractory TAK, duplicate patients, or unavailable data. Ultimately, 19 studies fulfilled our inclusion criteria and were included in this meta-analysis (13, 18–29, 36–41). A flow diagram of the literature search and selection is shown in **Figure 1**. Among the included studies, most came from Asia and Europe. Twelve studies were case series (19–21, 23, 25, 26, 29, 36, 37, 39–41) and seven were cohort studies or RCTs (13, 18, 22, 24, 27, 28, 38). Detailed characteristics for these individual studies are summarized in **Table 1**.

**Table 1:** Detailed characteristics of 19 included studies on tocilizumab in patients with refractory Takayasu arteritis.

Author	Year	Country	Study type	Patient source	Trial period	Number	Female	Age, y	Onset age, y	Disease duration, m	Diagnosis criteria	Previous DMARD	TCZ usage	TCZ duration, m	Follow-up, m
Ishii	2022	Japan	Case series	Tohoku University Graduate School of Medicine	2018-2020	34	28	NA	40.8 ± 15.3	150.0 ± 91.2	JCS2008/ACR1990	NA	NA	1.0-18.0	45.0
Alibaz	2021	Turkey	Cohort	10 tertiary centers in Turkey	NA	23	22	33.0 ± 12.4	26.7 ± 12.1	NA	ACR1990	csDMARD/TNFI	8 mg/kg/m iv or 162 mg/w ih	18.0 (1.0-81.0)	18.0 (1.0-81.0)
Campochiaro	2021	Italy	Case series	3 tertiary centers in Italy	NA	12	12	32.3 ± 12.8	NA	61.2 ± 44.4	ACR1990	csDMARD/TNFI	8 mg/kg/m iv or 162 mg/w ih	12	NA
Gon	2021	Japan	Case series	Kyoto University Hospital	2000-2017	5	5	31.2 ± 13.9	NA	NA	JCS2008/ACR1990	NA	8 mg/kg/m iv	24.0-53.0	NA
Isobe	2021	Japan	Case series	Sakakibara Heart Institute	2015-2019	19	17	41.4 ± 13.1	27.3 ± 15.5	171.6 ± 166.8	JCS2017	csDMARD	162 mg/w ih	NA	27.6 ± 14.4
Li	2021	China	Case series	First Affiliated Hospital of Anhui Medical University	2017-2020	11	10	NA	34 (19-46)	32.3	NA	csDMARD	8 mg/kg/m iv	NA	12
Mekinian	2021	France	Cohort	International multicenter study	2017-2019	118	101	NA	30 (8, 62)	27.6 (0, 372)	ACR1990/MIC	csDMARD	8 mg/kg/m iv or 162 mg/w ih	13.0 (1.0, 105.6)	30.0 (2.6, 104.4)
Prieto	2021	Spain	Cohort	26 referral centers in Spain	2014-2020	54	46	42.0 (32.5, 50.5)	NA	12.0 (I3.0, 31.5)	ACR1990/MIC	csDMARD/TNFI	8 mg/kg/m iv or 162 mg/w ih	NA	13.5 (6.0, 41.0)
Wu	2021	China	Case series	Zhongshan Hospital	2013-2020	33	27	26.0 (19.5, 35.5)	NA	14.0 (3.0, 49.0)	ACR1990	csDMARD	4-8 mg/kg/m iv	NA	12
Campochiaro	2020	Italy	Cohort	San Raffaele Hospital	2000-2018	17	16	39.8 ± 11.6	NA	152.5 ± 118.8	ACR1990	csDMARD	8 mg/kg/m iv or 162 mg/w ih	NA	NA
Kilic	2020	Turkey	Case series	Hacettepe University	to 2017	15	13	40.7 ± 12.1	35.4 ± 12.5	45.6 ± 43.8	ACR1990	csDMARD/TNFI	8 mg/kg/m iv	NA	16.1 ± 12.4
Kong	2018	China	Cohort	Zhongshan Hospital	2010-2016	9	8	32.1 ± 11.8	NA	10.0 (5.0, 43.0)	ACR1990	csDMARD4	8 mg/kg/m iv	6	6
Mekinian	2018	French	Case series	French Takayasu network	2009-2016	46	35	43.0 (29.0-54.0)	NA	NA	ACR1990/MIC	csDMARD/TNFI	8 mg/kg/m iv	NA	10.8 (6.0-24.0)
Nakaoka	2018	Japan	RCT	TAKT study	2014-2015	18	16	31.1 ± 18.1	NA	77.5 ± 88.4	JCS2008	NA	162 mg/w ih	4.8	12
Zhou	2017	China	Case series	Peking Union Medical College Hospital	2014-2016	13	12	27.9 ± 6.8	NA	34.7 ± 31.6	ACR1990	csDMARD/TNFI	8 mg/kg/m iv	NA	13.2 ± 3.8
Mekinian	2015	France	Cohort	French Takayasu network	2001-2013	14	NA	NA	NA	NA	NA	csDMARD	8 mg/kg/m iv	NA	24.0 (2.0-95.0)
Canas	2014	Colombia	Case series	ICESI University Medical School	2010-2013	8	8	27.8 ± 12.1	NA	8.1 ± 10.0	ACR1990	csDMARD/TNFI	8 mg/kg/m iv	18.0 (9.0-36.0)	18.5 ± 8.5
Goel	2013	India	Case series	Christian Medical College	NA	10	10	27.0 ± 13.2	NA	25.1 ± 20.0	ACR1990	csDMARD	8 mg/kg/m iv	6	NA
Tombetti	2013	Italy	Case series	San Raffaele Hospital	2004-2012	7	7	NA	26.6 ± 6.5	57.1 ± 31.6	ACR1990	csDMARD/TNFI	8 mg/kg/m iv	18.7 ± 11.1	18.7 ± 11.1

ACR, American college of rheumatology; csDMARD, Conventional synthetic DMARD; DMARD, Disease-modifying antirheumatic drug; ICESI, Instituto Colombiano Estudios Superiore de Incolda; JCS, Japanese Circulation Society; MIC, Modified ishikawa criteria; NA, Not available; TCZ, Tocilizumab; TNFI, Tumor necrosis factor-α inhibitor.

Data are expressed as mean or median or mean ± standard deviation or median (minimum-maximum) or median (25% interquartile range, 75% interquartile range).

### Baseline characteristics

A total of 466 patients with refractory TAK using TCZ were analyzed across 19 studies. Diagnosis of TAK was mainly based on the criteria of the American College of Rheumatology (ACR 1990) or the Japanese Circulation Society (JCS 2008). All patients had persistent active disease despite the use of standard care therapy. Sex distribution was predominantly female (88%, 95% CI 82-92%). The mean age at implementation of TCZ had a pooled estimate of 34.32 years (95% CI 30.39-38.23), and the mean age at symptom onset had a pooled estimate of 31.94 years (95% CI 27.68-35.19). The mean disease duration between onset and implementation was 64.68 months (95% CI 46.21-83.15). Type V (43%, 95% CI 34-53%) and type II (26%, 95% CI 17-37%) were the most common disease types according to the Numano classification, which meant that the involvement of the ascending aorta and aortic arch was the most prominent lesion characteristic. Before TCZ began, the mean NIH score and ITAS 2010 score were 2.64 (95% CI 2.21-3.06) and 6.68 (95% CI 5.30-8.05), respectively. Conventional synthetic DMARDs (csDMARDs) were used previously in 76% of patients (95% CI 55-89%), and tumor necrosis factor inhibitors (TNFIs) were used in 34% of patients (95% CI 16-59%). TCZ was usually administered 8 mg/kg every month intravenously or 162 mg every week subcutaneously, and combined csDMARDs were administered in 45% of patients (95% CI 34-58%). The pooled TCZ duration was 24.25 months (95% CI 16.92-31.57), and the follow-up period was 20.47 months (95% CI 16.42-24.53). A summary of patient-level information in the pooled results is presented in **Table 2**.

**Table 2:** Summary of patient-level information in the pooled results in this meta-analysis.

Variables	Number of studies	Total patients	Pooled estimates	95% CI	*I* ^2^, %
Female, %	18	452	0.88	0.82, 0.92	0.24
Age, y	14	282	34.32	30.39, 38.23	88.8
Oneset age, y	7	227	31.94	27.68, 35.19	74.80
Disease duration, m	14	367	64.68	46.21, 83.15	91.60
Numano classification					
I, %	8	235	0.19	0.09, 0.34	9.85
II, %	8	235	0.26	0.17, 0.37	2.22
III, %	8	235	0.04	0.01, 0.19	0.13
IV, %	8	235	0.03	0.02, 0.07	0
V, %	8	235	0.43	0.34, 0.53	0.50
NIH score	5	206	2.64	2.21, 3.06	88.90
ITAS2010 score	4	53	6.68	5.30, 8.05	71.90
Previous csDMARD, %	16	417	0.76	0.55, 0.89	36.58
Previous Anti-TNF, %	8	194	0.34	0.16, 0.59	17.01
Combined csDMARD, %	10	297	0.45	0.34, 0.58	3.67
TCZ intravenously, %	16	291	0.89	0.68, 1.00	93.55
TCZ duration, m	4	156	24.25	16.92, 31.57	63.80
Follow-up, m	10	317	20.47	16.42, 24.53	87.50
CRP					
Baseline, mg/L	14	384	29.04	23.16, 34.92	85.60
3m, mg/L	4	104	3.06	0.83, 5.29	91.30
6m, mg/L	8	271	1.66	0.97, 2.35	90.70
12m, mg/L	4	81	1.17	-0.18, 2.52	76.50
Last follow-up, mg/L	4	53	10.67	3.51, 17.84	91.10
ESR					
Baseline, mm/h	11	206	40.92	35.36, 46.47	68.70
6m, mm/h	5	107	7.48	4.08, 10.88	86.30
12m, mm/h	2	55	3.54	0.51, 6.58	87.40
Last follow-up, mm/h	4	53	10.2	5.26, 15.14	57.20
GC dose					
Baseline, mg/d	16	408	23.92	19.16, 28.67	94.20
3m, mg/d	4	104	11.79	8.12, 15.47	93.80
6m, mg/d	10	298	8.41	6.40, 10.43	93.10
12m, mg/d	6	118	6.26	4.24, 8.27	89.80
Last follow-up, mg/d	6	93	6.71	5.19, 8.23	65.50
Dose decrease, %	3	33	0.76	0.58, 0.87	0
Remission					
3m, %	5	115	0.45	0.36, 0.54	0
6m, %	8	260	0.80	0.63, 0.90	6.46
12m, %	5	90	0.79	0.69, 0.86	0
Last follow-up, %	3	33	0.73	0.43, 0.91	0.25
Relapse					
During TCZ-6m, %	3	40	0.22	0.09, 0.47	0.95
During TCZ-12m, %	6	83	0.17	0.05, 0.45	8.70
After TCZ discontinuation, %	4	30	0.18	0.02, 0.70	1.75
Discontinuation time, m	4	30	7.35	5.34, 9.35	83.3
Imaging progress					
6m, %	4	67	0.28	0.15, 0.46	0.80
12m, %	4	69	0.16	0.09, 0.27	0
Last follow-up, %	6	107	0.16	0.04, 0.42	12.97
Drug retention					
3m, %	3	57	0.89	0.74, 0.96	0.26
6m, %	5	113	0.75	0.46, 0.92	22.89
12m, %	6	114	0.68	0.50, 0.82	3.41
Last follow-up, %	10	228	0.69	0.51, 0.82	5.38
Adverse event					
Total adverse event, %	10	314	0.16	0.05, 0.39	23.92
Severe adverse event, %	5	173	0.04	0.02, 0.08	0
Infection, %	9	296	0.12	0.05, 0.28	18.87

CI, Confidence interval; CRP, C-reactive protein; csDMARD, Conventional synthetic DMARD; ESR, Erythrocyte sedimentation rate; GC, Glucocorticoid; ITAS2010, Indian Takayasu’s activity score 2010; NIH, National institutes of health; TCZ, Tocilizumab; TNFI, Tumor necrosis factor-α inhibitor.

### Clinical outcomes

#### Serological response

**Figure 2:**
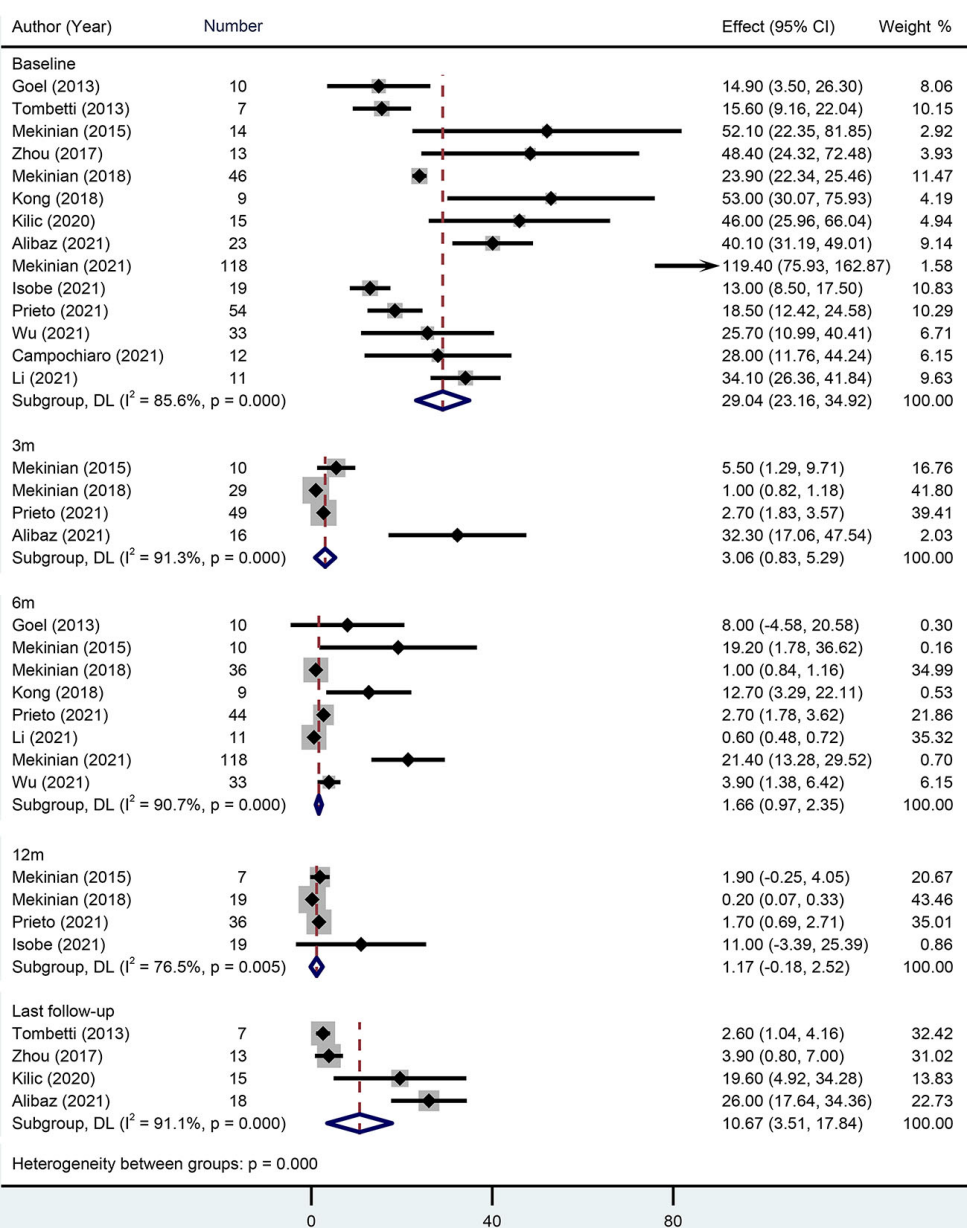
Forest plot showing the pooled results of CRP level at the baseline, 3-month, 6-month, 12-month and last follow-up.

Fourteen studies with 384 patients provided the baseline CRP when giving TCZ. The pooled baseline CRP was 29.04 mg/L (95% CI 23.16-34.92) with high heterogeneity (*I* ^2^ = 85.60%). During the follow-ups of 3 months, 6 months, and 12 months, pooled CRP levels were 3.06 mg/L (95% CI 0.83-5.29), 1.66 mg/L (95% CI 0.97-2.35), and 1.17 mg/L (95% CI -0.18-2.52), respectively. During the last follow-up, however, the pooled CRP reached up to 10.67 mg/L (95% CI 3.51-17.84) (**Figure 2**).

Twelve studies with 209 patients reported baseline ESR. Pooled baseline ESR was 40.92 mm/h (95% CI 35.36-46.47). During 6-month and 12-month follow-ups, pooled ESR levels were 7.48 mm/h (95% CI 4.08-10.88) and 3.54 mm/h (95% CI 0.51-6.58), respectively. At the last follow-up, however, the pooled ESR reached up to 10.2 mm/h (95% CI 5.26-15.14) (**Supplementary Figure 1**).

#### Clinical response

In terms of clinical response, GC dose, remission rate, and relapse rate were analyzed.

**Figure 3:**
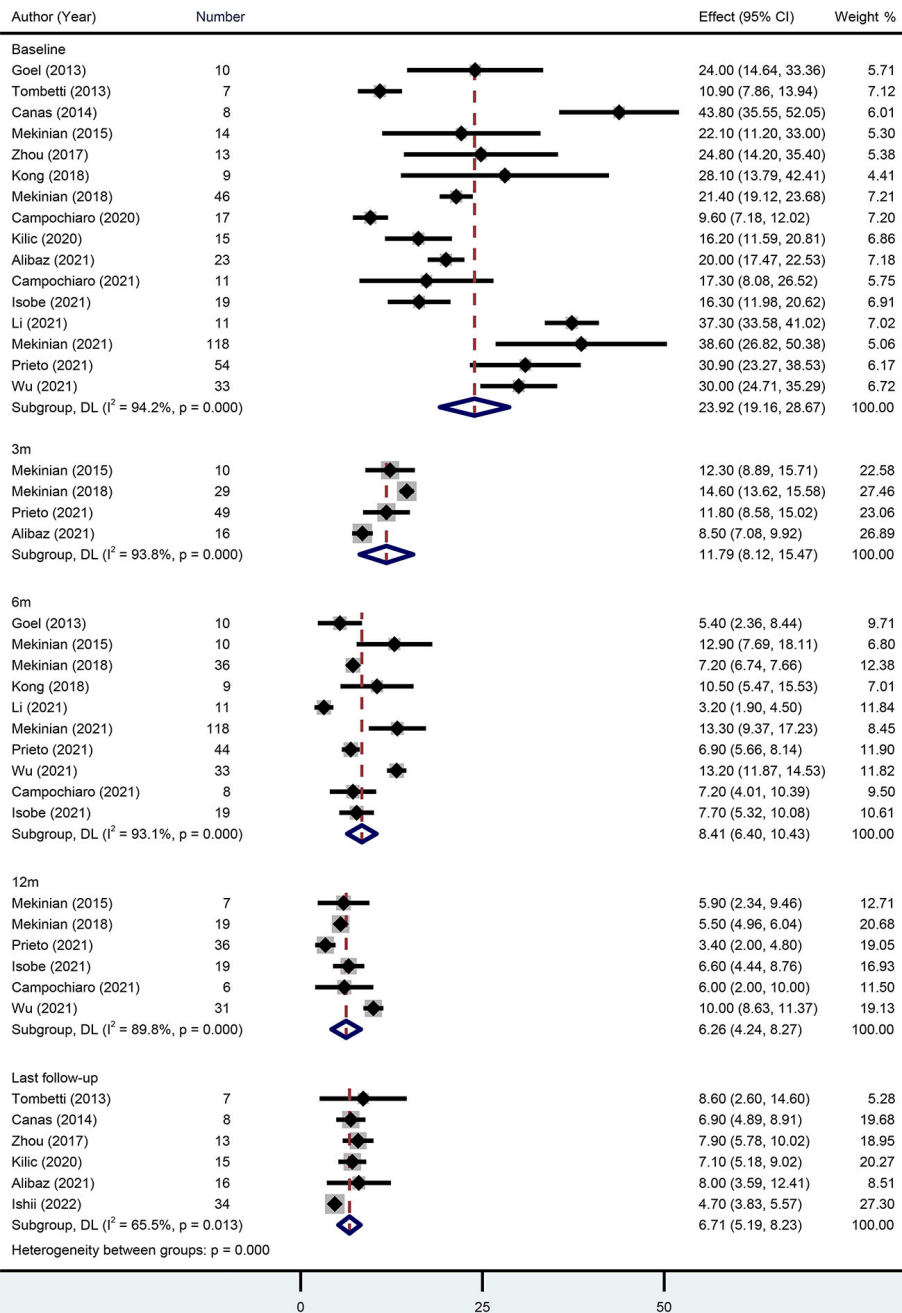
Forest plot showing the pooled results of glucocorticoid level at the baseline, 3-month, 6-month, 12-month and last follow-up.

When TCZ was initiated, baseline GC dose was reported in 16 studies with 409 patients. The pooled estimate was 23.92 mg/d (95% CI 19.16-28.67) with high heterogeneity (*I* ^2^ = 94.20%). At the 3-month, 6-month, 12-month, and last follow-ups, the pooled GC doses were 11.79 mg/d (95% CI 8.12-15.47), 8.41 mg/d (95% CI 6.40-10.43), 6.26 mg/d (95% CI 4.24-8.27), and 6.71 mg/d (95% CI 5.19-8.23), respectively. Approximately 76% (95% CI 58-87%) of patients achieved a GC dose decrease (**Figure 3**).

**Figure 4:**
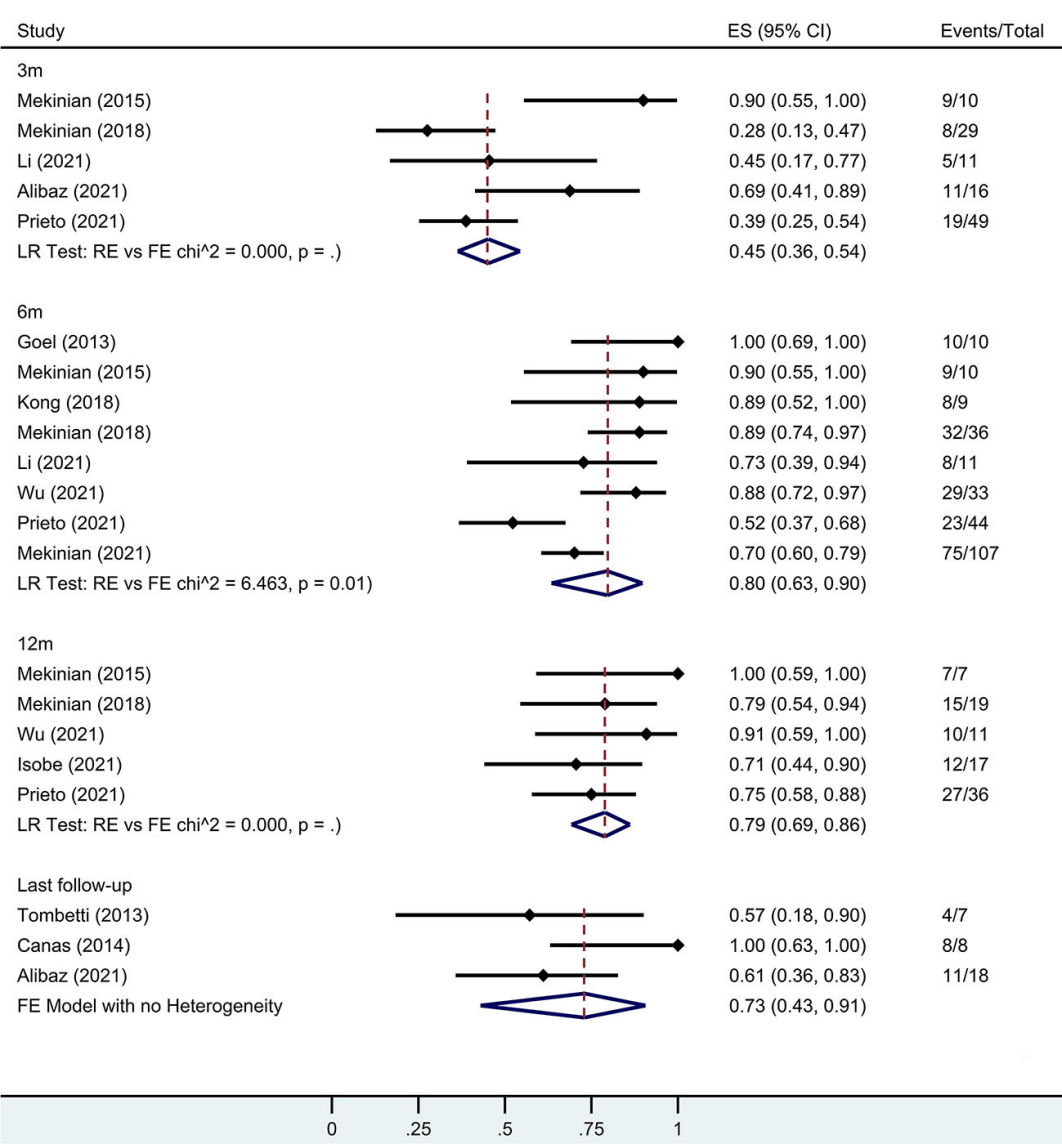
Forest plot showing the pooled results of remission rate at the 3-month, 6-month, 12-month and last follow-up. LR, Likelihood Ratio. RE, Random-Effect model. FE, Fixed-Effect model.

Several individual studies calculated remission rates during follow-up. At the 3-month, 6-month, 12-month, and last follow-ups, the rates were 45% (95% CI 36-54%), 80% (95% CI 63-90%), 79% (95% CI 69-86%), and 73% (95% CI 43-91%), respectively (**Figure 4**).

Seven studies with 117 patients reported 12-month relapse rates during TCZ. Pooled results showed that TAK patients under TCZ treatment had a relapse rate of 17% (95% CI 5-45%) during the 12-month follow-up. Four studies calculated relapse rates after TCZ discontinuation. The pooled estimate was 18% (95% CI 2-70%) when the TCZ discontinuation time was 7.35 months (95% CI 5.34-9.35).

#### Imaging response

Computed tomography angiography (CTA), magnetic resonance angiography (MRA), ^18^F-fluorodeoxyglucose-positron emission tomography (FDG-PET), and ultrasonography (US) were the most common imaging techniques for assessing the efficacy of TAK therapy. Most patients were evaluated as “improved” or “stable” on imaging; however, the rates of imaging progress were pooled as 28% (95% CI 15-46%), 16% (95% CI 9-27%), and 16% (95% CI 4-42%) at the 6-month, 12-month, and last follow-ups, respectively.

#### Drug retention

Two studies had a 6-month trial period and TCZ was stopped after the trial ended (37, 38). For patients taking medicine for a long time, TCZ retention rates were pooled as 89% (95% CI 74-96%) at 3 months, 75% (95% CI 46-92%) at 6 months, 68% (95% CI 50-82%) at 12 months, and 69% (95% CI 51-82%) at the last follow-up. Main reasons for TCZ discontinuation included inefficacy, adverse events, remission, and cost.

#### Adverse events

Regarding adverse events, data from 10 studies with 314 patients were analyzed. Adverse events occurred in 16% (95% CI 5-39%) of patients on the basis of pooled results. Specifically, infection was the most common adverse event, with a rate of 12% (95% CI 5-28%). Severe adverse events had a rate of 4% (95% CI 2-8%), including serious infection, major adverse cardiovascular events, and other adverse events leading to TCZ discontinuation.

## Discussion

The treatment option of refractory TAK is challenging. Most refractory TAK patients suffer GC-resistant events and frequent relapses after GC tapering, even when combined with csDMARDs (4, 5). TCZ, as a novel biologic agent of the anti-IL-6 receptor, selectively blocks the IL-6 signaling cascade, as a way to induce remission, decrease relapse, and reduce GC toxicity (42, 43). However, due to limited clinical data and experience, TCZ is not recommended for routine use in refractory TAK patients by either the EULAR or ACR guidelines (2, 3). Therefore, the evidence of all current literature on TCZ in refractory TAK patients urgently needs to be summarized.

Compared with previous systematic reviews focusing on the efficacy of biological agents in TAK (5, 44–46), the current study pays special attention to the baseline characteristics and clinical outcomes of TCZ in refractory TAK patients, with the largest sample size and the most included studies. It was found that TCZ treatment provided favorable outcomes in terms of steroid-sparing effects, clinical response, drug retention, and minimizing adverse effects for patients with refractory TAK.

Prior to TCZ usage, CRP and ESR were up to 29.04 mg/L and 40.92 mm/h, respectively, indicating active disease status in refractory TAK patients despite high-dose GC with adjunctive DMARD therapy. When receiving TCZ treatment, most patients presented significant decreases in inflammatory marker levels and GC doses. At the end of the 12-month follow-up, pooled CRP, ESR, and GC dose fell back to 1.17 mg/L, 3.54 mm/h, and 6.26 mg/d, respectively, achieving the target GC dose of ≤10 mg/day as recommended by the EULAR guideline (2). Interestingly, at the last follow-up (at approximately 20 months), CRP, ESR, and GC dose were slightly raised (**Supplementary Figure 2**), indicating new potential risk of the disease becoming active again. The above phenomenon was further confirmed by the remission rate: from 80% and 79% at the 6- and 12-month follow-ups, to 73% at the last follow-up. Two main reasons might contribute to these results. First, to reduce GC-related adverse events, GC exposure was limited when patients achieved remission, resulting in a fluctuation of disease activity. Second, some patients might develop resistance to TCZ treatment.

Disease remission and relapse were the two major concerns during TAK treatment. We found that TCZ treatment could provide favorable clinical outcomes, with a remission rate of 79% and a relapse rate of 17% during the 12-month follow-up. High-dose GCs, csDMARDs, and TNFIs, as the most common agents for achieving disease control, were compared with TCZ in a series of studies. For GCs, the TAKT study is the only RCT comparing the efficacy of TCZ *vs.* GC in patients with refractory TAK. The results showed that relapse occurred in 44.4% of TCZ-treated patients and 61.1% of GC-treated patients; however, no statistically significant difference between the two groups was found owing to limited sample size (18). In the open-label extension to 96 weeks, the final results indicated that TCZ provided a steroid-sparing effect and improvements on imaging evaluation (47). For csDMARDs, a retrospective cohort study compared 46 TCZ-treated patients and 46 age- and sex-matched csDMARDs-treated patients, and found that TCZ had a significantly lower cumulative incidence of relapse in refractory TAK patients (6.3% *vs.* 34.6%) (13). The results from another meta-analysis revealed remission and relapse rates of csDMARDs were 57.9% and 53.9% (5), inferior to the pooled results of TCZ in our review (79% and 17%). Although TCZ showed some signs, there was insufficient high-quality evidence to prove it superiority over csDMARDs. For TNFIs, several observational studies compared the efficacy of TCZ and TNFIs in patients with refractory TAK. Mekinian et al. found that the proportions of complete and partial remission rates and relapse-free survival were comparable for TCZ and TNFIs (13). In their latest multicenter retrospective study with 209 patients, the results still showed an equivalent relapse rate (27). Another multicenter comparative study also observed the similar results in clinical outcomes (24). However, more RCTs are warranted to investigate the efficacy and safety of TCZ and TNFIs in the future.

The pooled TCZ retention rate was favorable, up to 69% at the last follow-up. Inefficacy was the main reason for TCZ discontinuation, followed by adverse events, remission, and cost. However, the indication for discontinuation of TCZ due to remission is unclear. Several studies reported inconsistent results comparing the retention rates of TCZ and TNFIs. One study showed a comparable drug retention rate between TCZ and TNFIs (57% *vs.* 56%) (24), but another reported a significantly lower retention rate under TCZ treatment (41% *vs.* 67%) (22). The observed difference may come from the physician’s preference and a bias about TNFIs as the first-choice of biologic therapy in most TAK patients.

In this review, approximal 18% of patients experienced disease relapse after TCZ discontinuation, with a pooled discontinuation duration of 7.35 months. Wu et al. (21) reported that six out of fourteen cases experienced relapse after TCZ withdrawal, but no patient suffered from disease flare in another study (28). It seemed that prolonged TCZ treatment might help prevent disease relapse. At present, no guidelines make any recommendations on the best duration of TCZ treatment (2, 3). Further comparative studies concerning the effectiveness and duration of TCZ are needed.

It is worth noting that adverse events occur during TCZ treatment. A series of high-quality RCTs presented incidence rates of adverse events in other autoimmune diseases, including rheumatoid arthritis, polymyalgia rheumatica, and systemic sclerosis (48–50). The reported rates of adverse events and severe adverse events were 86-94% and 7-13%, respectively. In our pooled results, adverse events occurred in 16% of TAK patients. Infection proved the most common adverse event and a rate of 12%. Therefore, when administering TCZ therapy to refractory TAK patients, physicians should pay more attention to monitoring vital signs and observing potential symptoms of infection, such as fever, asthenia, rash, and elevated leucocyte count.

Because of accurate disease monitoring during GC tapering and a high risk of relapse during TCZ treatment, regular follow-up should be considered in all patients. A comprehensive disease activity assessment is needed based on a combination of clinical symptoms, laboratory investigation, and imaging examination. Imaging surveillance is regarded as mandatory (2, 3), because of the better ability to detect signs of vessel wall thickening, stenosis, or other active inflammation performance. CTA, MRA, and FDG-PET are the most popular imaging modalities used to distinguish persisting vascular inflammation and identify luminal abnormalities (51, 52). Nevertheless, there is no current consensus on the optimal frequency interval between imaging examinations. According to our experience, imaging surveillance might start every 6–12 months in the quiescent course and every 1–3 months (or more frequent) early in the active course.

Some studies have also aimed to evaluate the benefit of TCZ in non-refractory TAK patients (53, 54). The TOCITAKA study was the first prospective multicenter open-label trial to assess the long-term efficacy of TCZ in treatment-naive TAK patients (54). Thirteen patients were included, eleven (85%) of whom achieved TAK remission, and six of whom discontinued GCs after 6 months of TCZ therapy. During the 12-month follow-up after TCZ discontinuation, relapse occurred among five of the patients (45%). Another study from Yoshida et al. (53) comprised 14 active TAK patients with GC+TCZ, and 18 patients with GC or GC+csDMARDs. All patients achieved remission after initial therapy, however, GC+TCZ therapy had a significantly lower relapse rate during GC tapering (14.3% *vs.* 55.6%). The above findings revealed that TCZ seemed to be an effective alternative induction regimen for non-refractory TAK patients, but disease relapse after TCZ discontinuation and GC tapering were still an issue.

Several limitations were taken into account in this review. First, our findings were mainly based on case series or cohorts, which may limit the application of our findings to the actual population. Therefore, we only selected studies with more than five patients in an attempt to strengthen the robustness and representativeness of the results as much as possible. Second, individual participant data were not available from the included studies, which may bring some potential publication bias and insufficient evidence. Nevertheless, this study is the largest systematic review and meta-analysis for refractory TAK patients treated with TCZ and can provide better clinical guidance. Third, there were seven multicenter studies covering a long period of time from France, Japan, Turkey, Italy, Spain, and international cooperation (13, 18, 19, 24, 27, 28, 39), some of which had overlapping patient sources and trial periods with the included individual cohort. For example, data retrieved from San Raffaele Hospital in Italy by Campochiaro et al. (22) from 2000 to 2018 had potential duplications when compared with multicenter studies by Mekinian et al. from 2017 to 2019 (27). We made an effort to find and remove the overlapping data, however, we could not guarantee the removal of all duplicate data.

## Conclusion

In conclusion, this systematic review and meta-analysis supports the favorable outcomes of TCZ treatment in terms of inflammatory markers, steroid-sparing effects, clinical response, drug retention and minimizing adverse effects for patients with refractory TAK. More high-quality comparative studies are needed to explore the efficacy and safety of TCZ in the future.

## Data availability statement

The raw data supporting the conclusions of this article will be made available by the authors, without undue reservation.

## Author contributions

Conception and design: LK, YL, GY, QX. Data collection: LK, YL, ZL. Statistical analysis: LK, YL, BC. Writing the article: LK, YL, ZL, YZ. Critical revision of the article: BC, GY, QX. Final approval of the article: LK, YL, ZL, YZ, BC, GY, QX. Obtained funding: YL, GY, QX. Overall responsibility: GY, QX. All authors contributed to the article and approved the submitted version.
