# Supplementary material for: Systematic review and meta-analysis of the current literature on tocilizumab in patients with refractory Takayasu arteritis
Source: Front Immunol. 2023 Feb 8;14:1084558. doi: 10.3389/fimmu.2023.1084558 (PMC9945188; doi:10.3389/fimmu.2023.1084558)

**Supplementary** **Online Content**

**Supplementary Table 1. Detailed search strategies from Ovid database**

**Supplementary Table 2. Major components of the 20-criterion quality appraisal checklist**

**Supplementary Table 3. Results of quality assessment of case series by 20-criterion quality appraisal checklist**

**Supplementary Table 4. Results of quality assessment of cohort studies by Newcastle-Ottawa Scale**

**Supplementary Figure 1. Forest plot showing the level of ESR in patients with refractory Takayasu arteritis at the baseline, 6-month, 12-month and last follow-up**

**Supplementary Figure 2. Line charts showing the changes of the CRP, ESR and glucocorticoid levels during the follow-up**

**Supplementary Table I.** Detailed search strategies from Ovid database (Resource 1: Ovid MEDLINE(R) and Epub Ahead of Print, In-Process, In-Data-Review & Other Non-Indexed Citations, Daily and Versions(R), 1946 to July 11, 2022; Resource 2: Embase, 1974 to 2022 July 11; Resource 3: Cochrane Central Register of Trails, July 2022.

| 1 | Tocilizumab.af. | 30013 |
| --- | --- | --- |
| 2 | exp Tocilizumab/ | 21827 |
| 3 | IL-6.af. | 349912 |
| 4 | exp IL-6/ | 375029 |
| 5 | interleukin-6.af. | 23 |
| 6 | 1 or 2 or 3 or 4 or 5 | 512426 |
| 7 | arteritis.af. | 49436 |
| 8 | exp arteritis/ | 64342 |
| 9 | vasculitis.af. | 141498 |
| 10 | exp vasculitis/ | 229859 |
| 11 | 7 or 8 or 9 or 10 | 271138 |
| 12 | takayasu.af. | 14873 |
| 13 | 6 and 11 and 12 | 807 |
| 14 | limit 13 to English language | 766 |

**Supplemental Table II.** Major components of the 20-criterion quality appraisal checklist for assessing case series

| **Major components** | **Judgment** |
| --- | --- |
| 1. Was the hypothesis/aim/objective of the study clearly stated? | Yes, Unclear, No |
| 2. Was the study conducted prospectively? | Yes, Unclear, No |
| 3. Were the cases collected in more than one center? | Yes, Partially reported, No |
| 4. Were patients recruited consecutively? | Yes, Unclear, No |
| 5. Were the characteristics of the patients included in the study described? | Yes, Partially reported, No |
| 6. Were the eligibility criteria (i.e. inclusion and exclusion criteria) for entry into the study clearly stated? | Yes, Unclear, No |
| 7. Did patients enter the study at a similar point in the disease? | Yes, Unclear, No |
| 8. Was the intervention of interest clearly described? | Yes, Partially reported, No |
| 9. Were additional interventions (cointerventions) clearly described? | Yes, Unclear, No |
| 10. Were relevant outcome measures established a priori? | Yes, Partially reported, No |
| 11. Were outcome assessors blinded to the intervention that patients received? | Yes, Unclear, No |
| 12. Were the relevant outcomes measured using appropriate objective/subjective methods? | Yes, Unclear, No |
| 13. Were the relevant outcome measures made before and after the intervention? | Yes, Unclear, No |
| 14. Were the statistical tests used to assess the relevant outcomes appropriate? | Yes, Unclear, No |
| 15. Was follow-up long enough for important events and outcomes to occur? | Yes, Unclear, No |
| 16. Were losses to follow-up reported? | Yes, Unclear, No |
| 17. Did the study provided estimates of random variability in the data analysis of relevant outcomes? | Yes, Unclear or partially reported, No |
| 18. Were the adverse events reported? | Yes, Partially reported, No |
| 19. Were the conclusions of the study supported by the results? | Yes, Partially reported, No |
| 20. Were both competing interests and sources of support for the study reported? | Yes, Partially reported, No |

**Supplemental Table III.** Results of quality assessment of case series by 20-criterion quality appraisal checklist

| Author | Year | 1 | 2 | 3 | 4 | 5 | 6 | 7 | 8 | 9 | 10 | 11 | 12 | 13 | 14 | 15 | 16 | 17 | 18 | 19 | 20 |
| --- | --- | --- | --- | --- | --- | --- | --- | --- | --- | --- | --- | --- | --- | --- | --- | --- | --- | --- | --- | --- | --- |
| Ishii | 2022 | Y | N | N | Y | Y | P | Y | Y | U | Y | N | Y | Y | N | Y | U | U | Y | Y | Y |
| Campochiaro | 2021 | Y | N | Y | Y | Y | Y | Y | Y | U | Y | N | Y | Y | Y | Y | Y | U | Y | Y | Y |
| Gon | 2021 | Y | N | N | Y | Y | P | Y | Y | Y | Y | N | Y | Y | Y | Y | Y | U | Y | Y | Y |
| Isobe | 2021 | Y | N | N | Y | Y | Y | Y | Y | U | Y | N | Y | Y | Y | Y | Y | U | Y | Y | Y |
| Li | 2021 | Y | N | N | Y | Y | P | Y | Y | U | Y | N | Y | Y | Y | Y | Y | U | Y | Y | Y |
| Wu | 2021 | Y | Y | Y | Y | Y | Y | Y | Y | Y | Y | N | Y | Y | Y | Y | Y | U | Y | Y | Y |
| Kilic | 2020 | Y | N | N | Y | Y | Y | Y | Y | Y | Y | N | Y | Y | N | Y | Y | U | Y | Y | Y |
| Mekinian | 2018 | Y | N | Y | Y | Y | Y | Y | Y | Y | Y | N | Y | Y | Y | Y | Y | U | Y | Y | Y |
| Zhou | 2017 | Y | Y | N | Y | Y | Y | Y | Y | Y | Y | N | Y | Y | Y | Y | Y | U | Y | Y | P |
| Canas | 2014 | Y | N | N | Y | Y | P | Y | Y | Y | P | N | Y | Y | N | Y | Y | U | Y | Y | P |
| Goel | 2013 | Y | N | N | Y | Y | P | Y | Y | Y | Y | N | Y | Y | Y | Y | Y | U | Y | Y | P |
| Tombetti | 2013 | Y | N | N | Y | Y | Y | Y | Y | Y | Y | N | Y | Y | Y | Y | Y | U | Y | Y | P |

N, NO, not reported; P, Partially reported; U, Unclear; Y, Yes, fully reported.

Items 1 to 20 indicate 20 components of quality assessment for case series, shown in Supplemental Table II.

**Supplemental Table IV.** Results of quality assessment of cohort studies by Newcastle-Ottawa Scale

| **Author** | **Year** | **Representativeness of the exposed cohort** | **Selection**  **of the nonexposed cohort** | **Ascertainment of exposure** | **Outcome of interest was not present at start of study** | **Comparability of cohorts on the basis of the design or analysis** | **Assessment of outcome** | **Was follow-up long enough for outcomes to occur** | **Adequacy of follow-up of cohorts** | **Total score** |
| --- | --- | --- | --- | --- | --- | --- | --- | --- | --- | --- |
| Alibaz | 2021 | 1 | 1 | 1 | 1 | 2 | 1 | 1 | 1 | 9 |
| Mekinian | 2021 | 1 | 1 | 1 | 1 | 1 | 1 | 1 | 1 | 8 |
| Prieto | 2021 | 1 | 1 | 1 | 1 | 1 | 1 | 1 | 1 | 8 |
| Campochiaro | 2020 | 1 | 1 | 1 | 1 | 1 | 1 | 1 | 1 | 8 |
| Kong | 2018 | 1 | 1 | 1 | 1 | 0 | 1 | 1 | 1 | 7 |
| Nakaoka | 2018 | 1 | 1 | 1 | 1 | 2 | 1 | 1 | 1 | 9 |
| Mekinian | 2015 | 1 | 1 | 1 | 1 | 0 | 1 | 1 | 1 | 7 |

**Supplementary Figure 1.** Forest plot showing the level of ESR in patients with refractory Takayasu arteritis at the baseline, 6-month, 12-month and last follow-up


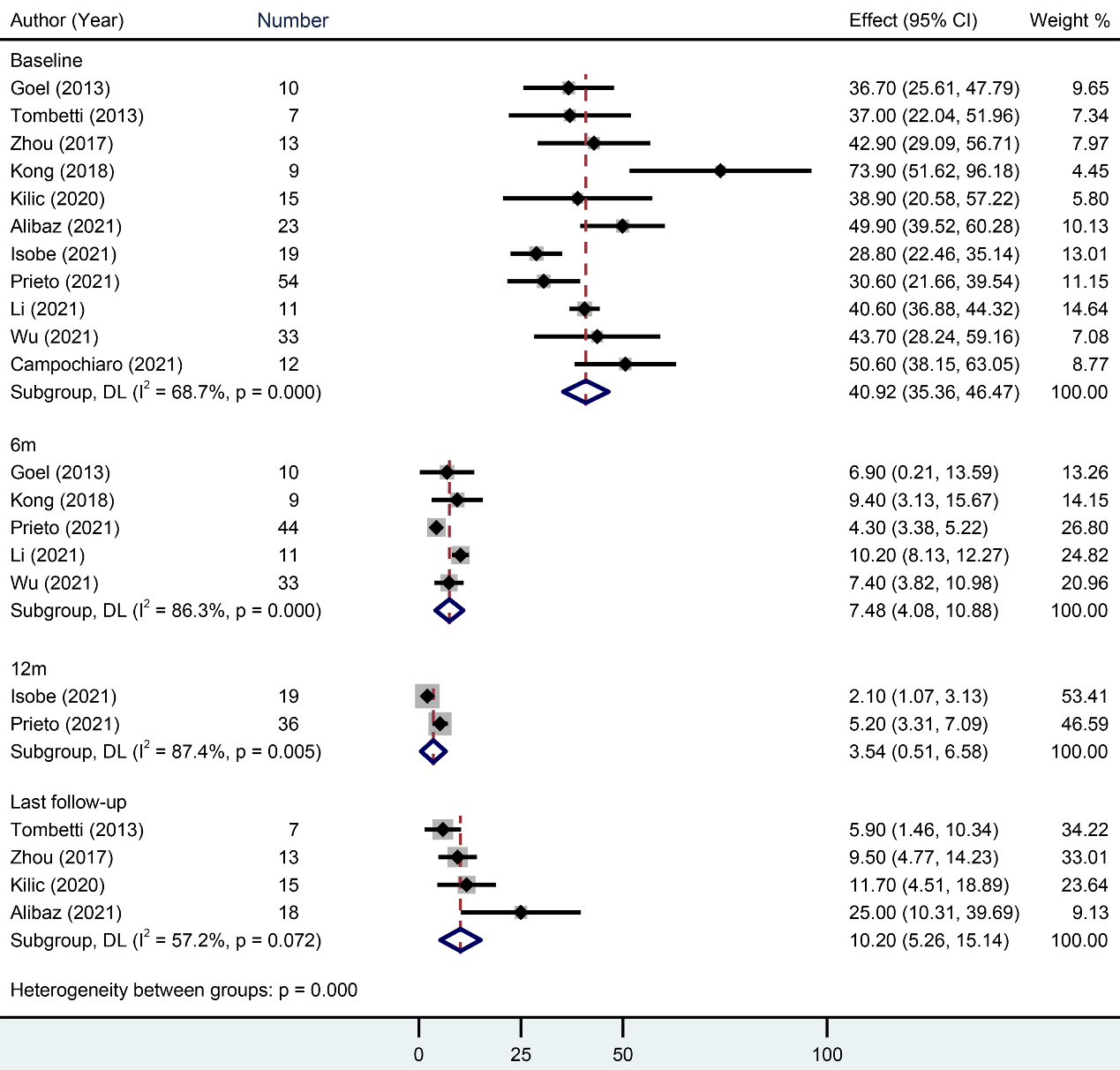


**Supplementary Figure 2. Line charts showing the changes of the CRP, ESR and glucocorticoid levels during the follow-up**


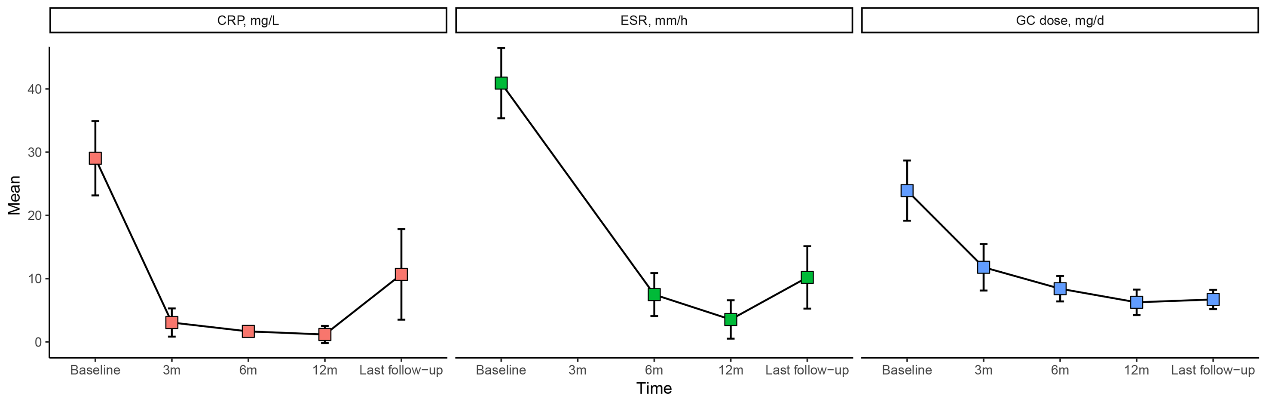

Supplement: Supplementary file 1 [file DataSheet_1.docx]
